# Fatal Motor Vehicle Crashes in Upstate and Long Island New York: The Impact of High Visibility Seat Belt Enforcement on Multiple Risky Driving Behaviors

**DOI:** 10.3390/ijerph20020920

**Published:** 2023-01-04

**Authors:** Joyce C. Pressley, Nirajan Puri, Tianhui He

**Affiliations:** 1 Departments of Epidemiology and Health Policy and Management, Columbia University, New York, NY 10032, USA; 2 Department of Biostatistics, Mailman School of Public Health, Columbia University, New York, NY 10032, USA

**Keywords:** seat belt, mortality, motor vehicle crash, traffic citation, New York, USA, speeding, alcohol use

## Abstract

Despite an observed daytime front-seat seat belt use that exceeds 90%, nearly half of motor vehicle occupants who die in New York State (NYS) each year are not wearing a seat belt. Crash outcomes were examined by occupant, vehicle, environmental and traffic enforcement patterns related to the annual *Click It or Ticket* high visibility seat belt enforcement campaign. Three periods of enforcement were examined: pre-enforcement, peri-enforcement (during/immediately after), and post-enforcement. Of the 14.4 million traffic citations, 713,990 (5.0%) were seat belt violations. Relative risk with 95% CI was assessed using deaths from the Fatality Analysis Reporting System (FARS) and SAS Glimmix 9.4 software. Mortality was lower peri-enforcement (32.9%) compared to pre- (40.9%) or post-enforcement (37.1%) (*p* < 0.001) and tended to be elevated in low enforcement response areas (43.6%). Fatalities were 30% lower (0.7, 95% CI 0.6–0.9) during peri-enforcement in models adjusted for demographics, law coverage, enforcement response, rural, weekend, impairment, speeding, and vehicle type. Adjusted mortality was higher in rural (1.9, 1.6–2.6), alcohol-involved (1.8, 1.4–2.9), and speeding-involved (2.0, 1.7–2.5) crashes. Peri-enforcement alcohol- and speed-involved fatalities tended to be lower in restrained, unrestrained and occupants missing belt status. The finding of lower mortality in both belted and unbelted occupant’s peri-enforcement—in the context of fewer fatal speed and alcohol-involved crashes—suggests that the mechanism(s) through which high visibility seat belt enforcement lowers mortality is through impacting multiple risky driving behaviors.

## 1. Introduction

Despite nearly five decades of preventive measures aimed at improving seat belt wearing, lack of use continues to be a leading factor in crash fatality for both the front and rear seats [1,2,3,4,5,6]. The National Highway Traffic Safety Administration (NHTSA) has piloted several initiatives, including a high visibility enforcement program known as *Click It or Ticket* to raise restraint (seat belt wearing) rates [6]. An early evaluation of an historic *Click It or Ticket* campaign in Elmira, New York raised observed seat belt wearing in that town to 80%, an historic milestone for the 1980′s [2]. Recent seat belt wearing observations conducted in adult front-seated motor vehicle (MV) occupants in New York State (NY) during the timeframe of this study demonstrated a daytime restraint use rate that is closer to 93% [7].

Despite accomplishing high daytime observed front-seat restraint rates, serious crashes, particularly fatal crashes, have demonstrated much lower restraint use in NY and nationally [3,8]. This uneven use of restraints is a contributing factor in many of the deaths of approximately 1100 New Yorkers each year. Although it is beyond the scope of this report, clinicians emphasize that another contributing factor to mortality is the post-crash treatment rendered. To address fatal clinical injury associated with restraint use and nonuse for adults injured in a motor vehicle crash, clinicians have noted the importance of raising awareness of the need for quick assessment and diagnosis, particularly with regard to thorax injuries [9,10,11,12]. As an example, the condition of isolated right atrial rupture with cardiac tamponade in MV crash occupants is a difficult, but important diagnosis and must be surgically corrected quickly [9]. In the setting of airbag deployment, there are reports of an association between seatbelt sign and cervicothoracic injury [11]. Steering wheel deformity is an independent predictor of serious, life threatening thoracic injury in drivers and front seat passengers and is associated with serious abdominal injury among front seated passengers. For these occupants, the risk of these injuries increases incrementally with increasing steering wheel deformity [12]. Driver’s who are not restrained experience traumatic brain injury and chest contusions with life threatening consequences more frequently than restrained occupants [13,14]. In high crash risk populations, restraint use can be used to decrease mortality and injury risk [15]. Crash and restraint information relayed to hospital staff by emergency personnel and law enforcement on the scene can provide clinicians with timesaving and lifesaving information which can be used to decrease mortality.

Mortality is only the tip of the iceberg as more than 160,000 injuries are attributed to MV crashes in NY State annually. On an average day, three New Yorkers die and 400 are injured in a MV crash [16]. Since many of these crash deaths and injuries occur at night, across the age span and in rear- as well as front-seated occupants, an ideal countermeasure would exert an impact across these areas as well. Previous evaluations of *Click It or Ticket* have measured daytime seat belt use generally in front-seated adults three weeks after the program’s high visibility enforcement period [17]. In addition, the evaluation of previous crash outcome data following the *Click It or Ticket* campaign was limited to daylight hours that more closely tracked the planned high visibility enforcement hours. Since the target hours and program design are not described fully in the publicity campaign, it is hypothesized that an effective high visibility field enforcement program will exert an impact on MV injury through multiple mechanisms. It is likely to impact across the occupant age span in both day and nighttime driving and, in addition to restraint status, possibly exert an impact across other risky driving behaviors, such as speeding and impaired driving.

This study extends previous analyses of the *Click It or Ticket* program by examining fatal crashes and ticketing pre-enforcement, peri-enforcement (during/immediately after) and post-enforcement periods. This study aims to describe factors impacting mortality and seat belt use across the three enforcement phases of this study (1) in both front- and rear-seat positions; (2) across the full age span; (3) across day and nighttime crashes; (4) by injury severity/mortality and seat belt status (restrained, unrestrained, missing restraint status). Percent change in county level ticketing during and post *Click It or Ticket* was compared to baseline, *Click It or Ticket* funding level by county and coverage by a restraint law.

## 2. Materials and Methods

### 2.1. Data Sources

The Fatality Analysis Reporting System (FARS) includes occupant, vehicle and crash characteristics of all fatal crashes occurring on a U.S. roadway. It is collected and distributed by NHTSA as a raw, public use data set for analytic use by motor vehicle safety researchers. FARS was used to analyze mortality, injury severity, restraint status, speed-related and alcohol-involved crashes in the 57 NYS counties included in this study [18,19]. Data on historic dates for implementation of the *Click It or Ticket* campaign were available from NHTSA.

Other publicly available data provided official *Click It or Ticket* funding levels at the municipal and county level, county characteristics, county enforcement funding and ticketing. Traffic ticketing and citation data were obtained from the NY State Department of Motor Vehicles (DMV) [20]. Traffic tickets included those issued to motorists for violations of: NYS Vehicle & Traffic Law (VTL), Thruway Rules and Regulations, Tax Law, Transportation Law, Parks and Recreation Regulations, Local New York City Traffic Ordinances, and NYS Penal Law pertaining to the involvement of a motor vehicle in acts of assault, homicide, manslaughter, and criminal negligence resulting in injury or death. Data extracted from the records of tickets on file with NYS DMV reflects the issuance of traffic tickets before adjudication. County-level roadway characteristics, such as roadway network composition, were extracted from the NYS Highway Mileage Report [21]. All county-level population and income characteristics were obtained from the United States Census Bureau. All county-level injury and mortality data were merged with a county-level analytic data set built by aggregating and then merging publicly available data sources [22]. These data sources contain MV traffic citations, transportation, socioeconomic variables, and roadway characteristics obtained from several publicly available NYS data sources.

### 2.2. Study Population

**Figure 1:**
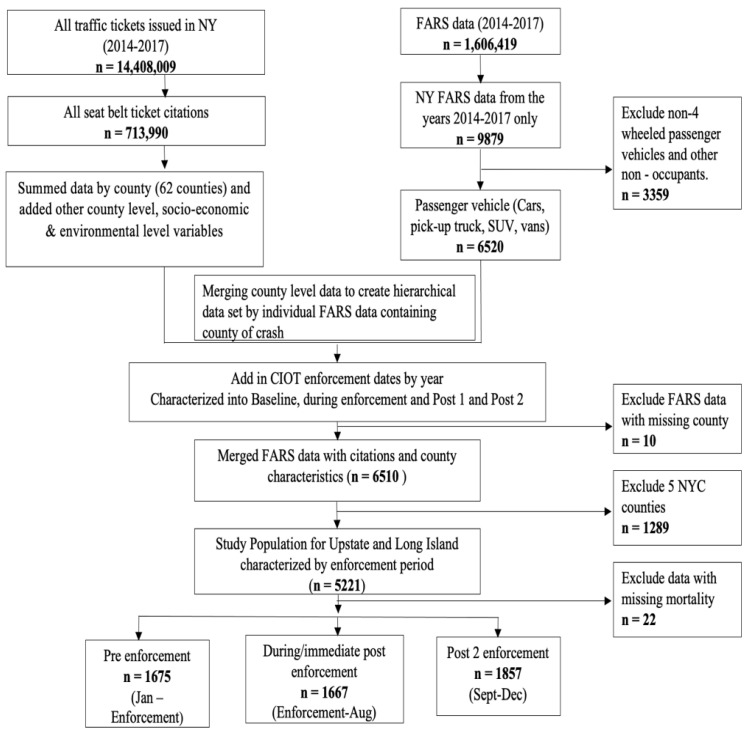
Study Population Flow Diagram. The inclusion and exclusion criteria employed to identify the study population from New York State (NYS) motor vehicle ticketing data and the Fatality Analysis Reporting System (FARS) is shown for the Upstate and Long Island regions of NYS.

The study population included MV occupants in four-wheeled passenger vehicles (*n* = 5199) who were involved in a fatal motor vehicle crash in Long Island and Upstate NY State over a four-year timeframe, 2014–2017 (Figure 1).

### 2.3. Variable Definitions

#### 2.3.1. Exposure

*Enforcement period*. Exposure was defined in three timeframes that captured enforcement period characteristics: pre-enforcement (January 1 up to the date of high visibility enforcement), peri-enforcement (Day 1 of high visibility enforcement for approximately 90 days) and post-enforcement (September 1 to December 31). Exact formal enforcement dates were: (1) 19 May–1 June 2014; (2) 18–31 May 2015; (3) 23 May–5 June 2016; (4) 22 May–4 June 2017. These timeframes were obtained using NHTSA public historic documents for dates of high visibility media announcements of upcoming enforcement periods and the actual dates of enforcement. Publicity consisted of national and local campaigns of both earned and paid media blitzes that preceded the actual initiation of high visibility ticketing.

#### 2.3.2. Outcome(s)

*Mortality*. Mortality and injury severity were ascertained across enforcement periods for each of the 4 years of the study using date of crash from FARS [18,19].

### 2.4. Enforcement Related Variables and Measures

*Enforcement*. Enforcement raw data were examined and graphed by month for each year of data across the 57 counties examined in this study. Seat belt violations comprised 5.0% of the 14.4 million moving violations. Pre-enforcement was defined as January 1st though the last date prior to enforcement initiation in May. The exact dates of high visibility enforcement varied slightly by year, but with high visibility enforcement always beginning in May, the peri-enforcement was defined as the first day of enforcement through 31 August. The post-enforcement period was 1 September through 31 December. Ticketing at baseline was calculated to enable the comparison of percent change in seat belt citations across enforcement periods. For monthly comparisons, the number of days in a month was standardized to 31 days. This allowed for the months of February, April, June, September, and November to be comparable to months that had 31 days of ticket exposure. Baseline average was the total seat belt citations in a month divided by the total number of months. Sensitivity analyses were performed using both three and four enforcement categories.

*Percent change in seat belt ticket enforcement*. The percent change increment in the citations for seat belt were calculated compared to baseline. There were 713,990 seat belt citations over the four-year timeframe of the study.

*Enforcement strength using county level categorization of percent change in ticketing during the enforcement period*. Counties were classified into three categories based on the percent increase in the seat belt ticket citation compared to their respective baseline: low, medium, and high. Counties in the *Low category* (N = 12 counties, mean 134.8 ± 67.53) included Chemung, Clinton, Essex, Franklin, Hamilton, Madison, Niagara, Schoharie, Steuben, Suffolk, Wyoming, Yates. Counties for the *Medium category* (N = 20 counties, mean 329.16 ± 50.74) included Albany, Allegany, Cattaraugus, Columbia, Cortland, Erie, Fulton, Genesee, Livingston, Monroe, Nassau, Oneida, Ontario, Orange, Orleans, Putnam, Schenectady, St. Lawrence, Wayne, Westchester. Counties in the *High category* (N = 25 counties, mean 517.82 ± 85.77) included Broome, Cayuga, Chautauqua, Chenango, Delaware, Dutchess, Greene, Herkimer, Jefferson, Lewis, Montgomery, Onondaga, Oswego, Otsego, Rensselaer, Rockland, Saratoga, Schuyler, Seneca, Sullivan, Tioga, Tompkins, Ulster, Warren, Washington. The Five (5) counties of NY City were excluded due to the presence of multiple Vision Zero initiatives and a highly established subway and public transportation system.

*County categorization by Click It or Ticket grant funds*. Two variables were used to assess enforcement resources. Counties were dichotomized by whether they received funding to enforce the *Click It or Ticket* campaign and the amount of funding per 100 county residents.

*Seat belt citations by county per 1000*. Restraint citations were calculated by taking the total seat belt citation in a county divided by the total population of that county multiplied by 1000. During the timeframe of this study, NY State law provides that the following persons wear a safety belt: (1) all front-seated occupants—driver and passengers; (2) passengers under age 16 years except when traveling in a vehicle for hire or a church van; (3) any passenger in any seating position being driven by a junior licensed driver. In addition, there were specific infant and children seat laws. Safety belt citations were obtained from the publicly available NYS DMV citation database and were included in the analysis of seat belt-related moving traffic citations. The proportion of seat belt-related citations per moving citation was calculated at the county level.

*Distribution of seat belt tickets by population by age group*. The proportion of the total county population included those who were aged 0–3 years, 4–7 years, 8–15 years, 16–17 years and 18 years and older. The infant and child ages correspond to seat belt and child restraint use laws. The first three age groups include occupants that are required to be restrained when traveling in a privately driven MV. The junior license driver age generally includes 16–17-year-olds.

*Moving citations*. Total moving violations included those issued between 2014 and 2017 (*n* = 14,408,009 citations) at the county level to NYS resident motorists aged 16–95 years. Moving violations included running red lights, stop signs, signals, lane changes, speeding, failure to yield right-of-way, driving while ability impaired (DWAI), violating interlock mandates and other citations issued for moving traffic law violations. Violations relating to registration, inspection, insurance, nonrestraint related equipment, parking and driver licensure were excluded from moving violations [20].

*Covered by a primary, secondary or no law*. During the time frame of this study, primary enforced law existed for: (1) front-seat passengers of all ages; (2) all persons in all seating positions if being driven by a junior driver; (3) all rear-seated passengers under the age of 16 years. In addition, several exemptions exist, most notably regarding vehicles for hire, which do not require seat belt use for any age group when seated in the rear seat.

### 2.5. Demographics

*Population per square mile*. Population density per square mile was obtained from census data for each county. The latest available census data were used to categorize counties by population size.

*Urban/Rural*. Urban and rural crash was obtained from a FARS database variable and from the county location.

*Rural-Urban Continuum Codes (RUCCs)*. RUCCs were available at the county level as a nine-category classification scheme with three metropolitan and six non-metropolitan categories. Non-metropolitan counties were classified by degree of urbanization and adjacency to a metropolitan area. The RUCCs allow counties to be classified into finer groups with the aim of capturing metropolitan influence on less urban areas [22,23].

### 2.6. Statistical Analysis

Demographic, restraint status, vehicle and crash characteristics and injury status were obtained at the individual level using FARS data from 2014–2017. County level demographic, economic and enforcement data were merged into the FARS data and were recoded using enforcement timeframes as defined using publicly available NHTSA *Click It or Ticket* enforcement dates specific to each year. Descriptive statistics were examined for traffic citations and county characteristics across the seat belt-related citations. All data were examined for numerical characteristics using bivariable analyses before being used in SAS logistic and Glimmix models [24]. Chi Square, logistic regression and multi-level logistic regression were used to analyze data. Our goal was to investigate characteristics of fatal crashes across different enforcement periods which is shown in the columns of our tables. Chi Square significance tests did not include small cell sizes associated with missing or unknown. SAS Glimmix was employed to control for the clustered, shared county level variables. All statistical analyses were two-sided and a *p*-value <0.05 was considered statistically significant. All analyses used SAS version 9.4 [25].

## 3. Results

*Study population*. The study population included 5199 occupants involved in a fatal motor vehicle collision during the 4 years spanning 2014–2017 (Figure 1). Population characteristics are shown aggregated for all occupants and separately for passengers and drivers. There were no significant differences in driver age or driver gender across the enforcement periods.

*Mortality*. Unadjusted mortality by population, vehicle and crash characteristics are shown in Table 1. Mortality differed significantly by age of the occupant, ranging from 38.0% in those less than age 20 to 55.5% in those aged 65 years or older. In the pediatric age range, mortality was significantly lower for those aged 0–7 years (12.2%) compared to those aged 8–15 years (16.7%), or those aged 16–19 years (23.2%).

*Front-* vs. *rear-seated occupants*. Passenger characteristics are shown by front and rear seating locations (Table 1). Front-seated passengers had significantly higher mortality than rear-seated passengers (Table 1). Both drivers and front-seated passengers had significantly lower mortality during the enforcement period than during the pre- or post-enforcement periods. Rear-seated passengers tended to have lower mortality both during the enforcement period (22.7%) and immediately post-enforcement (16.4%) than pre-enforcement (26.0%).

*Enforcement response*. Nearly one third of occupants involved in a fatal crash were in an area categorized as having a high enforcement response (Table 1). Proportionally, very few fatal crashes were in an area that had a low ticketing response compared to baseline (6.2%). The relationship between *Click It or Ticket* funding (in U.S. dollars) per 100 population was examined by the percent change in ticketing for seat belt violations during the enforcement month (May) compared to pre-enforcement for each county. The relationship of per capita dollars to percent change in ticketing was weak when viewed at the county level.

**Figure 2:**
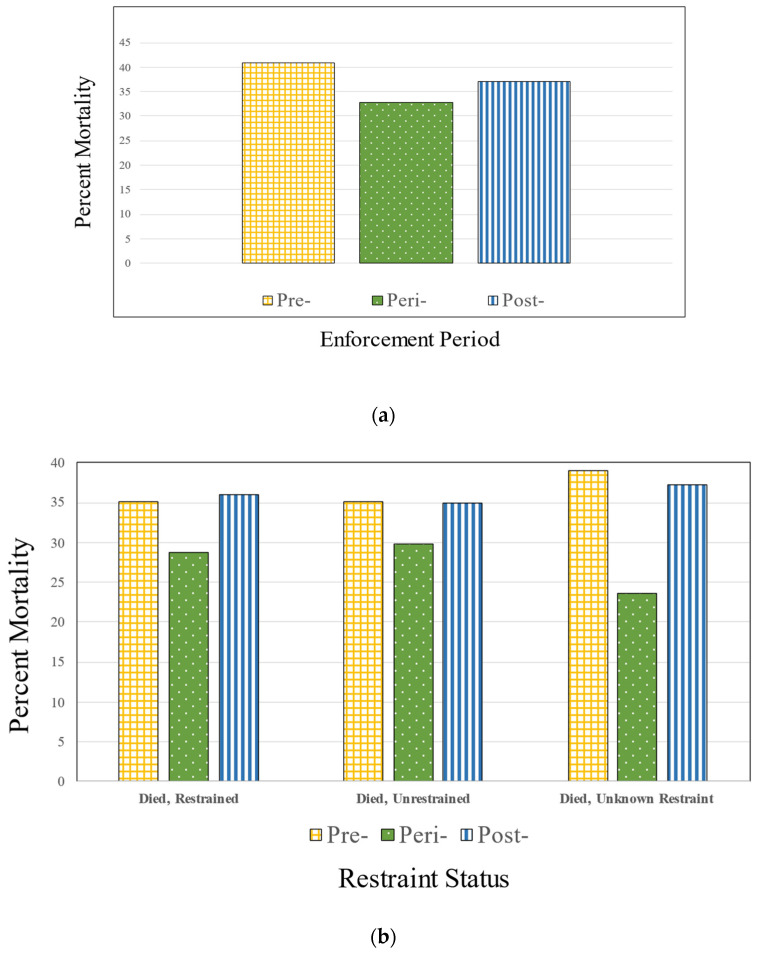

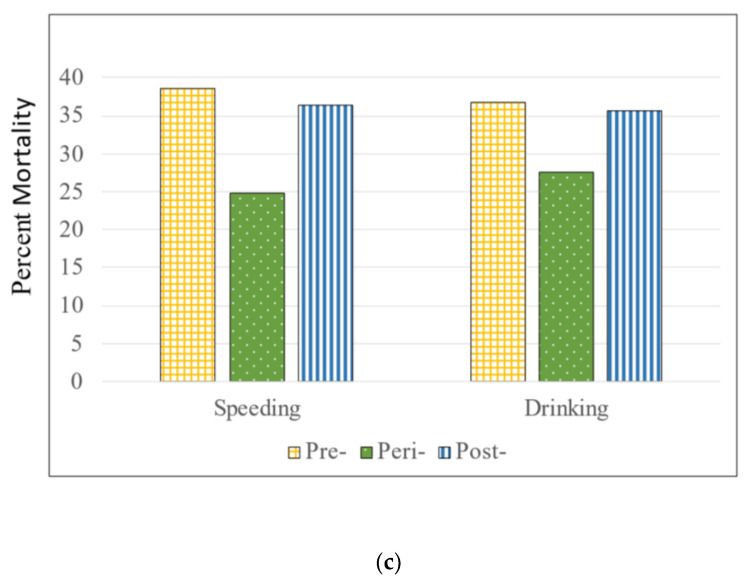
(**a**) Percent Mortality by High Visibility *Click It or Ticket* Enforcement Period. (**b**) Occupant Mortality by Restraint Status Period for Restrained, Unrestrained and Missing Restraint Status. (**c**) Proportion of Drivers in Fatal Crashes Who Were Speeding and/or Drinking by Enforcement Period.

*Restraint status*. Drivers had higher restraint use than passengers at baseline (76.4% vs. 72.5%, *p* = 0.001). Mortality in restrained passengers was less than half that of unrestrained passengers (Figure 2a). Mortality in restrained passengers was significantly lower during the peri-enforcement period than either during the pre- or post-enforcement period (*p* = 0.0012) and tended to be lower in those who were unrestrained during the peri-enforcement period compared to pre- or post-enforcement (*p* = 0.0598).

*Mortality*. Mortality was lower during the peri-enforcement period (32.9%) compared to pre-enforcement (40.9%) or post-enforcement (37.1%) (Table 1). Although mortality was highest in occupants in the lowest enforcement response areas (43.6%), this was not linear by level of enforcement strength as those in the medium response category had lower mortality than those in the high response areas (34.8% vs. 39.9%). Proportionally, fewer occupants died while being unrestrained during the peri-enforcement period than during either the pre-enforcement or post-enforcement timeframes. This was true across those who were restrained, unrestrained and missing restraint status (Figure 2b). Compared to the pre-enforcement period, fatal crashes involving speeding and alcohol were lower peri- and post-enforcement (Figure 2c).

*Mortality and vehicle characteristics*. Mortality was higher in passenger cars (43.5%) than in SUVs, vans or pickup trucks. Mortality decreased nearly linearly by increasing vehicle model year, from 53.9% for models older than 1994 to 26.1% for models 2015 or newer.

*Urban/rural status*. Mortality was significantly higher in rural crashes than in urban crashes with more than half (55.4%) of the study population with known urban/rural crash status being involved in a rural crash (not shown). There was no difference in the distribution of urban vs. rural crash locations across study periods.

*Time of day and day of week*. There were significantly fewer nighttime fatal crashes during the peri-enforcement period compared to either pre- or post-enforcement (*p* < 0.0001) (Table 2). There were no differences in weekday vs. weekend fatal crashes across study periods. Mortality was only slightly higher at night and was lower on weekends than weekdays (Table 2).

*Impaired and speed-related crashes*. The proportion of fatal crashes that involved impairment or speed was significantly lower during the peri-enforcement period (Table 2). Mortality was nearly doubled in speed-related crashes. Impaired and speed-related crash mortality was lower peri-enforcement than pre- or post-enforcement (Figure 2c).

*Predictors of mortality in fatal crashes*. In unadjusted analyses, the peri-enforcement period showed significantly lower mortality than pre-enforcement or post-enforcement (Table 3). In adjusted models, the peri-enforcement period continued to have lower mortality than pre-enforcement, but not in the post-enforcement timeframe. Adjusted mortality for unrestrained occupants was nearly six-fold that of restrained occupants. Occupants in vehicles that were speeding or being driven by a drinking driver had approximately three-fold higher mortality (Table 3). In adjusted models, occupant mortality for rural crashes was more than double that for urban crashes. Newer vehicles manufactured after 2008 showed a protective effect compared to model years 1994 and older (Table 3).

## 4. Discussion

This study demonstrates lower occupant mortality peri-enforcement compared to pre- and post-enforcement of the *Click It or Ticket* campaign in Upstate and Long Island regions in New York State. The finding of lower mortality during high visibility enforcement in both restrained and unrestrained occupants in combination with fewer speed- and alcohol-related crashes suggests that the mechanism(s) through which high visibility enforcement lowers mortality during *Click It or Ticket* is through impacting multiple behavioral risk factors associated with fatal crashes. This initially perplexing finding of lower mortality in both restrained, unrestrained, and missing restraint status motor vehicle occupants occurs in the context of fewer speed-related and alcohol-involved fatal crashes peri-enforcement compared to pre-enforcement and post-enforcement study periods.

This finding is consistent with anecdotal reports of law enforcement practices during the enforcement period that notes officers may initially observe a speeding vehicle which gives probable cause for a traffic enforcement stop. Excessive speed (10 or more miles over the posted limit or over 80 in a 65 MPH zone on interstate highway) is said to be the most frequent violation encountered [26]. Previous reports note that law enforcement stops during the high visibility enforcement periods are frequently for speeding or alcohol [26].

It is important to note that the inclusion criteria for this study population differs from that of prior evaluations of the *Click It or Ticket* program. In contrast to several other evaluations, this analysis included all crashes, day or night, and both front- and rear-seated occupants. Because the program aims to increase enforcement of belt wearing in front-seated, day-time occupants of four-wheeled passenger vehicles, previous studies tended to limit their crash and fatality evaluations to those crash times and conditions. However, since the limited hours of enforcement were not followed uniformly across jurisdictions and the hours of high visibility were not generally available to the public, it is hypothesized that drivers might be primed by seeing high visibility daytime enforcement and be more law abiding at night as well. In addition, the highest risk driving behaviors, lowest belt use and higher fatality tends to be at night [17].

This study was performed before passage of the current all age, all seating positions primary enforced New York State seat belt law. Prior to passage, New York had primary seat belt enforcement for private vehicles for: (1) drivers and all front-seated occupants; (2) occupants under age 16 years in all seating positions; and (3) occupants of all ages in all seating positions when driven by a junior licensed driver. Thus, at the time of this study, a vehicle could not be pulled for having an unrestrained, rear-seated adult passenger unless they were being driven by a junior licensed driver.

In 2017, NHTSA estimated that “seat belt usage saved an estimated 14,955 lives” [27]. With only 53% of fatalities in motor vehicle collisions being belted, NHTSA further estimates that an “additional 2549 lives could have been saved” by belt wearing. In studies that have evaluated belt use in the highest belt wearing states and compared it to the lowest belt wearing states, the strength of enforcement was key whether the law was primary or secondary [17]. This study further noted that the most important feature distinguishing the two groups was enforcement, primary vs. secondary, and not the state’s driver demographic composition or funds spent during the campaign on media blitzes. Assessing the full health and cost impact of seat belt law gaps—the impact of being covered versus not covered—and the role of primary vs. secondary enforcement of seat belt laws for both rear- and front-seated passengers is an area ripe for further studies as states grapple with legislation initiatives to close these gaps.

Counties with the lowest percent change in ticketing during the peri-enforcement period tended to have higher mortality during the enforcement period, but this relationship was not linear by enforcement strength as the counties categorized as having a medium *Click It or Ticket* response exhibited the lowest mortality during the enforcement period. Mortality was higher in rural counties. The finding that ticketing response was not closely correlated to the per capita dollars spent has been reported [17].

Approximately three decades ago, NYS had an observed seat belt wearing rate of approximately 49%. Its northern neighbor, Canada, had experienced some success in using linked publicity and enforcement campaigns to improve their seat belt wear rates. Piloting of similar programs in small and medium upstate NY cities produced dramatic improvements in seat belt-wearing, initially from 49% to 77% with supplemental programs the following year showing an observed belt use rate as high as 80% [2,28]. In 2017, New York’s observed front seat belt wearing was at 93% [29]. However, seat belt wearing is lower in fatal crashes, in nighttime crashes, in teens not covered by graduated driver licenses, in rear seated occupants, in taxis and vehicles for hire, in older vehicles, in those using alcohol or drugs and across geographical areas [1,30,31,32,33,34,35].

During this study timeframe, the national *Click It or Ticket* campaigns target front seat belt use in occupants deemed to be 15 years or older and are conducted in phases, typically lasting several weeks. The first phase is free earned media publicity at the national, state, and local levels, followed by paid media and then a two-week high-visibility enforcement period. Previous studies have noted that the campaigns have shown success with increased belt use being observed in at least 43 of 50 participating states and territories [6,17]. Of interest is that the NHTSA funded surveys for observation of seat belt wearing have been conducted approximately three weeks following the *Click It or Ticket* campaign. These seat belt wear rates are collected by state through contractors who use a standardized protocol to observe front seat occupants’ belt status. The belt status of rear seat occupants is not collected [6,17]. Although seat belt laws and enforcement of those laws can modify the risk of injury, restraint use remains lower for rear-seated than front-seated occupants, particularly in teens and adults [30].

This study differed from previous studies that were focused more narrowly on evaluating the *Click It or Ticket* program impact. This study focused more broadly on total motor vehicle mortality in four-wheeled passenger vehicles prior to, during/peri-campaign, and distally post campaign. A previous evaluation of the *Click It or Ticket* campaign limited their inclusion of fatal crashes in FARS data to daytime crashes (4 am until 8:59 pm) as well but included only front-seat outboard occupants of passenger vehicles, aged 15 years and older. The methods in this study differ from those of previous evaluations of *Click It or Ticket*, in that, all fatal crashes were analyzed—not just those occurring during the day.

While the program is aimed at the general driving population, NHTSA has supplemented this by funding programs that specifically targeted low belt use population subgroups, including some historical programs that previously targeted some of the rural areas in this study [36]. In addition, these findings and those of other investigators, could be useful in targeting high risk areas and focusing messaging on high-risk population subgroups [37]. Among states with secondary enforcement laws, those who achieved higher belt usage had higher levels of enforcement, with citations per capita that ranged from three to four times higher than secondary law states achieving lower belt usage. Although there is a case report in which the state’s belt use improvement was related to their media dollars expended, most states showed enforcement to be a more important factor in improving belt use rates [26]. One study that examined areas with and without additional enforcement demonstrated a 6.4% increase in belt wearing for every location where additional traffic enforcement was present [38].

This study has limitations. The analytic data set did not have information on crashes in which there was no fatality which prevents generalizing these findings to all road crashes. Further study using data that includes nonfatal crashes would increase the generalizability. The analytic data set grouped funding and enforcement at the county level. It is possible that enforcement varied across incorporated areas within a county and that disparities and inequity in enforcement existed within a county and/or across counties. Funding for enforcement varied across jurisdictions and was multi-sourced. We had funding information only for the source dedicated to be spent on the Click It or Ticket program. Counties and jurisdictions had multiple sources of baseline funding for enforcement, such as tax-based funding, which varied between poorer and richer counties. There are reports that many counties lacked enforcement funding outside of the actual time frame allotted for high visibility. The FARS data set has missing and under reported data on alcohol and drug-impaired driving. Model years of vehicles were examined, but the analytic data set did not contain specific information on differences in safety features or safety ratings across vehicles. Although time periods of high visibility enforcement were available, the actual dates these were observed may have varied across counties and jurisdictions. Lastly, this study did not include the five counties comprising New York City because of the initiation of multiple Vision Zero initiatives.

Automated enforcement and technology advances, such as seat belt reminders and alcohol sensors, may improve the disparities observed over the three phases of this study. In addition to the numerous improvements in vehicle structure, design, handling, automatic emergency breaking features, lane keeping and other safety features that are being touted for their protective effects in motor vehicle injury, it is possible that vehicle age may be associated with higher mortality through, for example, the presence of safety belt reminders that are present in newer, but absent in older, vehicles.

Further study is needed to assess whether and to what degree and under what circumstances automated camera enforcement can narrow inequities and lower dangers associated with enforcement. While it looks to be the future of enforcement for many traffic laws and is currently being touted as a tool toward creating a safer, more equitable roadway, its impact has not been evaluated for restraint use or impaired driving.

## 5. Conclusions

In conclusion, the findings of this study suggest that the mechanism(s) through which high visibility enforcement lowers mortality is through impacting multiple risky driving behaviors associated with fatal crashes. These findings demonstrate the value of high visibility enforcement, highlight rural program disparities and provide clues where fatal MV crashes might be lowered further. It is worth noting that the *Click It or Ticket* program was suspended in many geographic areas during the pandemic. Further study is needed to assess whether, and to what degree, suspension of high visibility enforcement was a contributing factor in the pandemic associated increase in motor vehicle mortality [39].

## Figures and Tables

**Table 1:** Population characteristics of fatal crashes in the Upstate and Long Island regions of New York State by *Click It or Ticket* Enforcement Periods, FARS 2014–2017.

Variable	Pre-Enforcement	Peri-Enforcement	Post-Enforcement	Total	(χ^2^ *p*-Value)
Total (*n*, %)	1675 (32.22)	1667 (32.06)	1857 (35.72)	5199	
Occupant injury severity ^1^					
Fatal injury	685 (40.90)	548 (32.87)	689 (37.10)	1922	<0.0001
Serious Injury	191 (11.40)	190 (11.40)	193 (10.39)	574	
No apparent/minor injury	799 (47.70)	929 (55.73)	975 (52.50)	2703	
Occupant fatal injury restraint status ^1^					
Restrained	394 (23.52)	323 (19.38)	403 (21.70)	1120	0.8972
Unrestrained	202 (12.06)	171 (10.26)	201 (10.82)	574	
Unknown	89 (5.31)	54 (3.24)	85 (4.58)	228	
Driver Characteristics (*n* = 3437)					
Driver age (years) ^1^					
15–19	65 (5.79)	76 (7.34)	80 (6.25)	221	0.5994
20–44	496 (44.21)	481 (46.47)	572 (44.69)	1549	
45–64	322 (28.70)	270 (26.09)	356 (27.81)	948	
65 and older	238 (21.21)	207 (20.0)	268 (20.94)	713	
Unreported	1 (0.09)	1 (0.10)	4 (0.31)	6	
Driver sex/gender					
Male	757 (67.47)	707 (68.31)	858 (67.03)	2322	0.8269
Female	364 (32.44)	327 (31.59)	419 (32.73)	1110	
Driver belt status ^1^					
Restrained	832 (74.15)	791 (76.43)	951 (74.30)	2574	0.8286
Unrestrained	172 (15.33)	160 (15.46)	206 (16.09)	538	
Unknown	118 (10.52)	84 (8.12)	123 (9.61)	325	
Driver injury severity					
Fatal injury	516 (45.99)	398 (38.45)	531 (41.48)	1445	0.0035
Serious injury	98 (8.73)	82 (7.92)	104 (8.13)	284	
No apparent/minor injury	508 (45.28)	555 (52.62)	645 (50.39)	1708	
Driver fatal injury restraint status^1^					
Restrained	305 (27.18)	247 (23.86)	315 (24.61)	867	0.8237
Unrestrained	138 (12.30)	108 (10.43)	151 (11.80)	397	
Unknown	73 (6.51)	43 (4.15)	65 (5.08)	181	
Passenger characteristics (*n* = 1725)					
Passenger age (years)					
15 and younger	96 (17.71)	149 (24.07)	108 (19.15)	353	0.0247
15–19	82 (15.13)	108 (17.45)	91 (16.13)	281	
20–44	212 (39.11)	197 (31.83)	218 (38.65)	627	
45–64	81 (14.94)	76 (12.28)	61 (10.82)	218	
65 and older	71 (13.10)	89 (14.38)	86 (15.25)	246	
Passenger belt status ^1^					
Restrained	365 (67.34)	449 (72.54)	406 (71.99)	1220	0.0555
Unrestrained	137 (25.28)	140 (22.62)	107 (18.97)	384	
Unknown	40 (7.38)	30 (4.85)	51 (9.04)	121	
Passenger injury severity					
Fatal Injury	167 (30.42)	148 (23.60)	158 (27.57)	473	0.0695
Serious Injury	93 (16.94)	106 (16.91)	85 (14.83)	284	
No apparent/minor injury	289 (52.64)	373 (59.49)	330 (57.59)	992	
Passenger fatal injury restraint status ^1^					
Restrained	89 (16.21)	76 (12.12)	88 (15.36)	253	0.3001
Unrestrained	64 (11.66)	63 (10.04)	50 (8.73)	177	
Unknown	14 (2.55)	9 (1.44)	20 (3.49)	43	
Seating position ^1^					
Front	1447 (86.39)	1360 (81.58)	1637 (88.15)	4444	<0.0001
Rear	208 (12.42)	273 (16.38)	208 (11.20)	689	
Unknown	20 (1.19)	34 (2.04)	12 (0.65)	66	

^1^ Chi-Square test performed without missing or unknown data.

**Table 2:** Roadway, Vehicle, and Crash Characteristics of Fatal Crashes in the Upstate and Long Island Regions of New York State by *Click it or Ticket* Enforcement Periods, FARS 2014–2017.

Variable	Pre-Enforcement	Peri-Enforcement	Post-Enforcement	Total	(χ^2^ *p*-Value)
Total (*n*, %)	1675 (32.22)	1667 (32.06)	1857 (35.72)	5199	
Crash-level characteristics (*n* = 5199)					
Rural crash ^1^					
Rural	737 (44.00)	707 (42.41)	788 (42.43)	2232	0.8525
Urban	589 (35.16)	553 (33.17)	644 (34.68)	1786	
Unknown	349 (20.84)	407 (24.42)	425 (22.89)	1181	
Day/night ^1^					
Day	872 (52.06)	1143 (68.57)	829 (44.64)	2844	<0.0001
Night	801 (47.82)	524 (31.43)	1026 (55.25)	2351	
Unknown	2 (0.12)	0 (0.0)	2 (0.11)	4	
Weekday/weekend					
Weekday	1135 (67.76)	1099 (65.93)	1270 (68.39)	3504	0.2761
Weekend	540 (32.24)	568 (34.07)	587 (31.61)	1695	
Ejected ^1^					
Totally	32 (1.91)	37 (2.22)	29 (1.56)	98	
Partially	83 (4.96)	77 (4.62)	64 (3.45)	224	
No	1557 (92.96)	1551 (93.04)	1763 (94.94)	4871	0.1030
Unknown	3 (0.18)	2 (0.12)	1 (0.05)	6	
Rollover ^1^					
Yes	221 (13.19)	267 (16.02)	233 (12.55)	721	0.0073
No	1454 (86.81)	1399 (83.92)	1624 (87.45)	4477	
Unknown	0	1 (0.06)	0	1	
Speed-related crash ^1^					
Yes	423 (25.25)	280 (16.80)	376 (20.25)	1079	<0.0001
No	1238 (73.91)	1372 (82.30)	1466 (78.94)	4076	
Unknown	14 (0.84)	15 (0.90)	15 (0.81)	44	
Impaired-related crash ^1^					
Yes	288 (17.19)	202 (12.12)	244 (13.14)	734	<0.0001
No	1376 (82.15)	1453 (87.16)	1607 (86.54)	4436	
Unknown	11 (0.66)	12 (0.72)	6 (0.32)	29	
Manner of collision ^1^					
Non-collision	849 (50.69)	695 (41.69)	853 (45.93)	2397	<0.0001
Rear end	110 (6.57)	145 (8.70)	154 (8.29)	409	
Head on	243 (14.51)	300 (18.00)	385 (20.73)	928	
Angle	417 (24.90)	453 (27.17)	417 (22.46)	1287	
Sideswipe—same direction	30 (1.79)	35 (2.10)	25 (1.35)	90	
Sideswipe—opposite direction	11 (0.66)	11 (0.66)	20 (1.08)	42	
Other/not reported	15 (0.90)	28 (1.68)	3 (0.16)	46	
Vehicle model year ^1^					
1994 and under	34 (2.03)	32 (1.92)	36 (1.94)	102	<0.0001
1994–1997	66 (3.94)	40 (2.40)	83 (4.47)	189	
1998–2004	562 (33.55)	529 (31.73)	550 (29.62)	1641	
2005–2008	420 (25.07)	349 (20.94)	457 (24.61)	1226	
2009–2011	195 (11.64)	234 (14.04)	234 (12.60)	663	
2012–2014	286 (17.07)	315 (18.90)	288 (15.51)	889	
2015–2018	112 (6.69)	168 (10.08)	203 (10.93)	483	

^1^ Chi-Square test performed without the missing or unknown data.

**Table 3:** Unadjusted and Multivariable Adjusted Analysis of Fatal Motor Vehicle Crashes on a U.S. Roadway, Fatality Analysis Reporting System (FARS), 2014–2017.

Variables		Unadjusted OR (95% CI)	*p*	Adjusted OR (95% CI)	*p*
Age		1.0 (0.999–1.001)	0.6967	1.033 (1.018–1.048)	<0.0001
Sex/gender	Male	1.0 (ref)			
	Female	0.706 (0.628–0.794)	<0.0001		
Enforcement Period	Per	1.0 (ref)		1.0 (ref)	
	Peri	0.708 (0.615–0.815)	<0.0001	0.715 (0.560–0.912)	0.007
	Post	0.853 (0.745–0.976)	0.8248	0.865 (0.689–1.086)	0.2122
Restraint status	Restrained	1.0 (ref)		1.0 (ref)	
	Unrestrained	3.922 (3.376–4.557)	<0.0001	5.837 (4.403–7.739)	<0.0001
Speeding	Not speeding	1.0 (ref)		1.0 (ref)	
	Speeding	3.162 (2.754–3.630)	<0.0001	3.152 (2.415–4.113)	<0.0001
Drinking	Not drinking	1.0 (ref)		1.0 (ref)	
	Drinking	2.902 (2.473–3.405)	<0.0001	2.711 (1.979–3.712)	<0.0001
Enforcement response strength	Low	1.0 (ref)			
	Medium	0.692 (0.550–0.872)	<0.0001		
	High	0.862 (0.678–1.096)	0.6523		
Vehicle model year	Less than 1994	1.0 (ref)		1.0 (ref)	
	1994–1997	0.864 (0.533–1.399)	0.0003	0.859 (0.355–2.081)	0.7368
	1998–2004	0.611 (0.409–0.913)	0.0381	0.534 (0.256–1.113)	0.0940
	2005–2008	0.550 (0.366–0.825)	0.7639	0.523 (0.250–1.094)	0.0853
	2009–2011	0.433 (0.284–0.660)	0.0072	0.386 (0.181–0.825)	0.0140
	2012–2014	0.349 (0.231–0.529)	<0.0001	0.316 (0.149–0.672)	0.0028
	2015–2018	0.302 (0.195–0.468)	<0.0001	0.279 (0.129–0.607)	0.0013
Weekday	Weekday	1.0 (ref)		1.0 (ref)	
	Weekend	0.822 (0.728–0.928)	0.0016	0.731 (0.587–0.910)	0.0051
Time	Night	1.0 (ref)			
	Day	0.970 (0.867–1.087)	0.6027		
Rural/urban	Urban	1.0 (ref)		1.0 (ref)	
	Rural	1.825 (1.598–2.084)	<0.0001	2.238 (1.840–2.722)	<0.0001

## Data Availability

We used publicly available data for this study which is available at the links provided in the reference section. The methods section describes the data available and the reference section the link where it can be obtained. Although publicly available, we do not have permission to redistribute the data.
